# Intelligent outcome measures in liaison psychiatry: essential even if not desirable: Commentary on … a multidimensional Framework for Routine Outcome Measurement in Liaison Psychiatry (FROM-LP)^†^

**DOI:** 10.1192/pb.bp.115.053397

**Published:** 2016-08

**Authors:** George Tadros

**Affiliations:** 1 Aston Medical School, Aston University and Birmingham and Solihull Mental Health Foundation Trust

## Abstract

Service development is guided by outcome measures that inform service commissioners and providers. Those in liaison psychiatry should be encouraged to develop a positive approach that integrates the collection of outcome measures into everyday clinical practice. The Framework for Routine Outcome Measurement in Liaison Psychiatry (FROM-LP) is a very useful tool to measure service quality and clinical effectiveness, using a combination of clinician-rated and patient-rated outcome measures and patient-rated experience measures. However, it does not include measures of cost-effectiveness or training activities. The FROM-LP is a significant step towards developing nationally unified outcome measures.

We have firmly moved on from a time when service provision was planned largely to meet a demand for specific unmet needs and practice that was deemed to be safe was considered to be good enough. We have now entered a new era in which current practice has been dominated by outcome measures demanded by a multitude of commissioners and monitoring organisations. Some clinicians resent the whole notion of outcome measures as abomination and some accept them as part of the modern, forced reality, others view it as an opportunity to improve practice, develop evidence for the effectiveness of their work and promote their specialties. The right combination of academic approach, clinical expertise and managerial support seems to be essential for this kind of forward thinking.

Though this is true to all specialties, it poses a great challenge to psychiatric service providers in particular owing to the nature of our work. There are multiple factors that could influence our patients' journey and outcomes arising from prevention to recovery: times of crisis, relapses and need for social and family support. This challenge is even greater and more difficult to subjugate in liaison psychiatry, owing to the added layers of complexity caused by the influence of physical comorbidity. The nature of health provision in the acute hospital, usually focusing on a physical health agenda, the urgency in crisis presentations, the difficulty of proving the singular effect of liaison psychiatry in the midst of many other parallel initiatives, the reliance on external pathways for patient management and the existence of individual and organizational stigma towards patients with mental health issues create a complex picture when attempting to examine outcomes.

Intelligent outcome measures should provide a balance between three main domains: performance (especially response time), service quality and cost-effectiveness. Focusing only on one aspect can be misleading and unhelpful for future planning. Liaison psychiatry has a variety of roles in urgent and emergency psychiatric crisis within acute trusts. These roles could have influence on every aspect of acute hospitals' performance, including staff skills, with implications on service provision and patient life beyond hospital walls. This commentary will examine the utilisation of outcome measures in quantifying the effectiveness of liaison psychiatric services with critique for the use of the FROM-LP as proposed by Peter Trigwell and colleagues.^1^

## Performance and rapid response

The ability to provide a rapid response to a request for psychiatric assessment has become an essential criterion for successfully operating liaison teams, mainly because of increasing scrutiny of the accident and emergency department 4-hour targets^2^ and a desire to reduce length of stay on in-patient wards.^3^ Rapid response can have a very positive effect on patient journey and outcome measures. The timing of psychiatric consultation is an essential factor in determining a patient's length of stay in hospital,^4–6^ especially when psychosocial assessment is completed in the first few days of admission to a general hospital ward.^7,8^ The difficultly is that patients presenting with more severe psychiatric symptoms, such as suicidal ideation, florid psychosis or behavioural problems, tend to receive more timely consultations than those with less demanding presentations. Consequently, patients with less obvious psychiatric symptoms have a tendency to either go undetected or wait for a long time before being referred to liaison psychiatry for assessment,^9^ which can lead to poorer outcomes.^10^ To achieve the best outcome, identifying patients who need psychiatric assessment as early as possible, through an effective triage system, is as important as responding rapidly to a referral for liaison psychiatry. The factors associated with delayed referral to a psychiatry liaison team usually include stigma,^11–13^ lack of mental health training and skills, unclear referral pathways, perceived lack of efficacy to change patient outcomes, and patient's refusal of a psychiatric assessment. The presence of physical illness has been found to delay the identification of psychiatric disorders and hinder referrals to psychiatric liaison teams.^14^ On the other hand, rapid response has been associated with higher level of acute hospital staff satisfaction with service provision.^15^

The Framework for Routine Outcome Measurement in Liaison Psychiatry (the FROM-LP) clearly identifies performance as a priority, focusing on recording outcomes for patients using the Identify and Rate the Aim of the Contact model. This would be essential in demonstrating the inwards and outwards referrals as well as the services and support offered. For the good reasons identified above, the FROM-LP also collects information regarding response time, whether for a single contact or a series of contacts. However, there is no attempt to quantify the length of time taken before mental health issues are detected and the patient is referred to the liaison team. It is a missed opportunity to encourage proactive work to promote early referral and more accurate detection.

## Quality service

Although performance-based data are important to measure activities, they would not mean much unless they bring quality to the services patients receive through those activities. Liaison psychiatry teams should always aim to improve quality of care that is provided to patients with physical and psychiatric comorbidities, as about 27% of patients admitted to medical wards have mental illness fulfilling DSM-IV criteria.^16^ Another layer of quality improvement would be achieved through up-skilling acute hospital staff to manage patients with psychiatric manifestations. Reports suggest that staff attitudes towards patients who attend hospital for reasons other than physical health may be negative,^11^ mainly because of lack of training,^17^ stigma^18,19^ and perceived difficulties in managing such patients' complex needs in an environment that is designed mainly for acute medical illnesses.^20,21^ This is particularly applicable to older persons, especially those with dementia.^22^ Staff up-skilling could be achieved through direct and indirect training as well as joint case-working. Research suggests that education can help to both eliminate discrimination of those with mental illness and up-skill acute hospital staff.^23^ The Rapid Assessment Interface and Discharge (RAID) service in Birmingham attributed a significant portion of their cost savings to supporting and training staff to manage patients who have not been referred for liaison psychiatry (RAID influence group).^24^ This sort of quality improvement usually leads to an increase in the number of referrals through enhanced detection of mental illness.

The FROM-LP clearly measures service quality and clinical effectiveness using a combination of clinician-rated outcome measures (CROMS), patient-rated outcome measures (PROMS) and patient-rated experience measures (PREMS). It provides a good description of clinical improvement from the clinician's outlook. In addition, it offers a variety of satisfaction measures from the patient, friends and family, and the referrer perspectives.

The FROM-LP offers an array of service quality outcome measures that are appropriate to liaison psychiatry, easy to administer and create measurable data. There is currently no attempt to measure training activities and their outcomes, nor to identify patients whose care quality has improved indirectly through the work of a liaison psychiatry team. Unfortunately, the FROM-LP authors offered some condition-specific assessment scales which are not related to outcome measures and some of them have already been updated by their authors, for example Addenbrooke's Cognitive Examination (ACE-R), or are inappropriate for older people, for example the Alcohol Use Disorders Identification Test (AUDIT-C).

## Cost-effectiveness

Having established that a well-operating liaison team would perform well with rapid response and would deliver valuable quality to patient care, it is still desirable to measure the team's cost-effectiveness for the purpose of future commissioning, which in the current climate is frequently a team survival need and a lever to improve service funding. However, it could occasionally lead to more pressure and a negative effect, especially if a team is compared with better funded teams or expected to achieve some unrealistic or specific cost savings in a short period of time.

Evidence for cost-effectiveness has been frequently established for specific liaison models or services such as hip fracture.^25^ The RAID model in Birmingham has been extensively evaluated, first internally,^24^ then by an NHS-Confederation-commissioned independent evaluation,^25^ which estimated that the cost/return ratio was £1: £4. More recently, an independent evaluation commissioned by the regional commissioning support unit^26^ showed similar savings: cost/return ratio of £1: £2.97. This was achieved through reducing admissions via accident and emergency, length of stay and re-admissions for in-patient groups. The majority of savings come from working with older people, especially those with dementia.^24,26^ This is despite the fact that two-thirds of hospital beds are occupied by elderly patients.^27^ Older patients on acute wards who experience lengthy admission and delayed discharges tend to develop anxiety regarding discharge destination, and report low mood, frustration, anger, disappointment and feeling disempowered.^28,29^ With increasing numbers of patients with dementia (850 000 according to the latest census (www.alzheimers.org.uk/statistics)), working with this group of patients becomes essential to fully achieving the saving potential.^24,25^ Hence the stress on good-quality dementia care in general hospitals in the government's National Dementia Strategy.^30^ Intelligent outcome measures should provide an encouraging framework for older adult liaison work.

Measuring cost savings provides a further challenge because of the complexity and variations of individual patients' features and the known paucity of clinical information and diagnostic coding concerning retrospective patient groups with mental health issues in general hospitals. There is no easy way to collect cost-saving data as it usually requires sophisticated statistical analysis and computer modelling. Nevertheless, it is essential that liaison teams are prepared for cost-effectiveness scrutiny. Intelligent outcome measures should collect data that would help with measurement of cost-effectiveness, such as illustration of work done to avoid admission, length of stay when admitted, discharge destination, rate of readmission at 28 days and 90 days, and breaches at the emergency department. The FROM-LP does not prompt for cost-saving data collection.

## Challenges in using outcome measures in liaison psychiatry

Most liaison psychiatry team members would like to consistently gather data to demonstrate the value that they hold in patient care and their journey through acute hospitals. However, unless electronic patient records are developed to accommodate data collection for outcome measures, the whole concept might be seen as time-consuming, despite its necessity. This perception leads to low response rate and a lot of missing data, which could hinder future analysis power. Most of the suggested CROMS, PROMS and PREMS rely heavily on clinician's and patient's subjectivity, which could lead to unavoidable bias. Moreover, patients' and referrers' satisfaction, or lack of it, could be a reflection of other components of the patient's journey or outcome that is not directly related to liaison psychiatry.

RAID services use an outcome form (RAID Discharge Outcome Form; https://raidnetwork.org/content/resources), which has been developed as part of patient electronic records, to improve compliance and reduce duplication. This electronic outcome form has its own weaknesses and the same inherited subjectivity flaws; however, it could complement the FROM-LP, especially in the cost-effectiveness domain. Nevertheless, there is a real need to have nationally agreed, consistent outcome measures for liaison psychiatry that would allow data combination and comparison for further research and future developments.

## Conclusions

Outcome measures are essential for clinical teams to evaluate their work, show their effectiveness and plan for future development. Measuring outcomes in a scientifically robust fashion is generally difficult in psychiatry but particularly challenging in liaison psychiatry. The the FROM-LP, using a combination of CROMS, PROMS and PREMS, provides a very useful framework. It is now readily available and helpful in measuring team performance and clinical quality, but it fails to measure delay in time from admission to referral to the liaison psychiatry service, which usually has negative effect on length of stay. It equally fails to collect data related to cost-effectiveness. In addition, it does not measure training activities which could have great significance in improving care quality and outcomes.

As subjectivity and bias are still strong barriers to overcome, there is a great need to develop independent measures. Until we succeed in developing electronic digital solutions for outcome measures, as part of patient records, clinicians will continue finding it difficult to comply with filling in forms for outcome measures in addition to simultaneously conducting full clinical and risk assessments and updating patient records.
